# Long‐Term Population Trends and Spatial Redistribution of Shorebirds at a Critical Migratory Stopover

**DOI:** 10.1002/ece3.74183

**Published:** 2026-09-02

**Authors:** Stephanie Feigin, Theo Diehl, Lawrence Niles, Amanda Dey, Joanna Burger

**Affiliations:** ^1^ Wildlife Restoration Partnerships Greenwich New Jersey USA; ^2^ Division of Life Sciences Rutgers University Piscataway New Jersey USA; ^3^ NJ Department of Environmental Protection Endangered and Nongame Species Program Trenton New Jersey USA

**Keywords:** conservation, migratory shorebirds, population trends, red knot, Ruddy turnstone

## Abstract

Understanding population change in migratory shorebirds is critical for conservation, particularly at key stopover sites where individuals concentrate during migration. Using nearly four decades of standardized peak aerial count data (1986–2025), we examined long‐term population dynamics of four abundant shorebird species at the Delaware Bay spring stopover: Red Knots ( *Calidris canutus rufa* ), Ruddy Turnstones ( *Arenaria interpres* ), Sanderlings ( *Calidris alba* ), and Semipalmated Sandpipers ( *Calidris pusilla* ). First, we evaluated species‐specific trends in peak abundance to distinguish interannual variability from sustained population declines. Log‐linear regression analyses for each species showed a statistically significant decline in Red Knot and Ruddy Turnstone populations from 1986 to 2025 (*p* < 0.001), whereas trends for Sanderlings and Semipalmated Sandpipers showed declines from historic numbers, but the declines were not statistically significant (*p* > 0.5). Focusing on the two significantly declining species, we assessed patterns of shoreline use within Delaware Bay. Red Knots showed a substantial shift in shoreline use within the bay over time, concentrating on the New Jersey (eastern) shoreline over time, with more than 85% utilizing the New Jersey shoreline from 2019 to 2025, while Ruddy Turnstones continued to use both shorelines, with values fluctuating between 35% and 65% across the time series. By integrating long‐term abundance trends with patterns of shoreline use, we demonstrate how long‐term monitoring is essential for interpreting population trajectories and informing conservation management at critical migratory stopover sites.

## Graphical abstract

Long‐term population monitoring at critical migratory stopover sites is essential for detecting population declines and informing conservation management. Using nearly four decades of standardized peak aerial count data (1986–2025), we examined population trends and spatial patterns of stopover use for four abundant shorebird species at the Delaware Bay spring stopover, finding statistically significant declines in rufa Red Knots and Ruddy Turnstones but not in Sanderlings or Semipalmated Sandpipers. Additionally, Red Knots exhibited a pronounced spatial redistribution toward the New Jersey shoreline over the study period, highlighting how integrating abundance trends with spatial data can reveal nuanced shifts in habitat use that are critical for targeting conservation actions at key stopover sites.

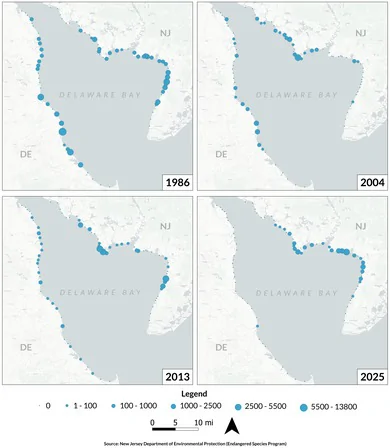

## 1 Introduction

Worldwide, shorebirds are among the most rapidly declining groups of birds, facing a wide range of threats throughout their annual migratory cycle (Andres et al. 2013; Morrison et al. 2001; Smith et al. 2020, 2023). Many species undertake exceptional migrations, often spanning hemispheres, making population persistence dependent on the availability and quality of breeding, wintering, and stopover habitats (Andres et al. 2013; Morrison et al. 2001). For example, some Red Knots undertake round‐trip migrations that can exceed 30,000 km annually (Baker et al. 2004; Duijns et al. 2017; Morrison et al. 2001; Niles et al. 2008). Across their ranges, shorebirds are exposed to habitat loss, prey limitation, predation, human disturbance, contaminants, extreme weather events, sea‐level rise, and climate change, with impacts that may act independently or cumulatively across sites (Galbraith et al. 2002, 2014, Goss‐Custard et al. 2006, Burger and Gochfeld 2016). Since migratory connectivity links population performance across geographically distant sites, understanding where and how population change occurs remains a central challenge in shorebird population ecology (Conklin et al. 2010; Marra et al. 2015).

Stopover sites are particularly critical within migratory networks because they provide the food and spatial resources necessary for birds to complete migratory flights. Changes in the quality or use of stopover habitats can therefore have disproportionate effects on population dynamics that can often be difficult to detect without long‐term monitoring (Smith et al. 2020, 2022, 2023; Galbraith et al. 2014; Burger, Dey, and Niles 2024). Delaware Bay is one of the most important spring stopover sites for migratory shorebirds in the Western Hemisphere, supporting hundreds of thousands of birds during the northward migration each May (Senner and Howe 1984, Burger 1986; Niles et al. 2008; Smith et al. 2022). Historically, the bay hosted up to 1.5 million shorebirds stopping over during their northbound migration to their Arctic breeding grounds (Niles et al. 2013, Senner and Howe 1984, Burger 1986). At the same time, millions of horseshoe crabs make their way to the Delaware Bay beaches to breed. During spring migration, shorebirds have a narrow window of time to stopover and double in weight before the next leg of their migration. For this reason, these eggs provide an important food source for many species in the bay, but most importantly, are a vital resource to migrating shorebirds who rely on this high‐quality food source to nearly double their weight in 2 weeks or less, prior to continuing their migration to Arctic breeding grounds (Baker et al. 2004; Botton et al. 1994; Niles et al. 2008; Burger, Porter, and Niles 2024). Since the 1990s, however, Horseshoe crabs have been harvested commercially and exploited for use as bait in conch and eel fisheries and before that for use as fertilizer (ASMFC 2009). In the late 1990s a sharp increase in horseshoe crab harvest from around 100,000 in the early 1990s to 2.5 million in 1998 resulted in a major decrease in the crab population and egg densities in the bay (Vision 2016). Historically, egg densities on most Delaware Bay beaches exceeded 100,000 eggs/m^2^ (Botton et al. 1994), but by 2000 had declined to fewer than 5000 eggs/m^2^ and have remained near that level since (Smith et al. 2022). Subsequently, shorebird populations that relied on crab eggs also crashed, with Red Knot counts declining from a peak of 94,460 in 1989 to < 50,000 by 1999 and about 13,000 by 2004, remaining below 20,000 on average since (Clark et al. 1993; Niles et al. 2008, ASMFC 2009; Vision 2016; USFWS 2014a, 2014b, 2016; Smith et al. 2023).

Among the 28 declining shorebird species in North America, the four most reliant on Delaware Bay, Red Knots ( *Calidris canutus rufa* ), Ruddy Turnstones ( *Arenaria interpres* ), Sanderlings ( *Calidris alba* ), and Semipalmated Sandpipers ( *Calidris pusilla* ), have experienced some of the steepest declines (45%–50%; Smith et al. 2023). These trends and the crash of the horseshoe crab population in Delaware Bay contributed to the federal listing of the Red Knot as Threatened in 2015, prompting continued annual monitoring of shorebird population trends and condition, horseshoe crabs, egg densities, and the Delaware Bay ecosystem (Baker et al. 2004; Duijns et al. 2017; Smith et al. 2020; USFWS 2014a, 2014b, 2016).

These declines have led to Delaware Bay becoming the focus of sustained conservation and management action following the population crash of shorebird species that are most reliant on the bay during migration. Following the horseshoe crab population crash, federal and state agencies implemented coordinated harvest management beginning in 1998, with adaptive management frameworks designed to support recovery of both horseshoe crabs and the shorebirds that depend on them (ASMFC 1998, 2009; McGowan et al. 2011). On the New Jersey side of the bay, beach restoration projects beginning in 2012, many in response to Superstorm Sandy, have rebuilt over a mile of degraded shoreline to support horseshoe crab spawning and shorebird foraging (Niles et al. 2013; Smith et al. 2020). At the same time, 12 New Jersey beaches are protected annually with seasonal closures, signage, and stewards to reduce human disturbance during the stopover period (Burger and Niles 2013; Burger, Dey, and Niles 2024). These interventions provide both a context for interpreting long‐term trends and a set of tools that conservation outcomes can be measured.

Long‐term monitoring at Delaware Bay has generated one of the most extensive shorebird datasets globally and has played a central role in documenting population change and informing management and conservation action along the Atlantic Flyway (Niles et al. 2008; Smith et al. 2020, 2023). Despite the availability of long‐term data, two questions remain poorly resolved at Delaware Bay: how species‐specific abundance trends have unfolded over multi‐decadal timescales, and whether long‐term declines are accompanied by shifts in spatial distribution within the stopover site. Annual peak counts have provided a valuable index of relative abundance at Delaware Bay (Clark et al. 1993; Niles et al. 2008; Smith et al. 2023), changes in peak counts may reflect not only population‐level change, but a redistribution of birds within the bay in response to shifting habitat conditions.

In this study, we examined long‐term population dynamics of four migratory shorebird species, Red Knots, Ruddy Turnstones, Sanderlings, and Semipalmated Sandpipers, using nearly four decades of annual count data from Delaware Bay to provide information on trends on declining species and provide data for management. We first evaluate species‐specific trends in peak abundance to distinguish interannual variability from sustained population declines. We then focus on Red Knots and Ruddy Turnstones, the two species exhibiting statistically significant long‐term declines and the most complete datasets, to assess whether changes in peak counts are accompanied by spatial redistribution between the New Jersey and Delaware shorelines. By integrating long‐term abundance trends with patterns of shoreline use, we demonstrate how long‐term monitoring is essential for interpreting population trajectories and informing conservation management at critical migratory stopover sites.

## 2 Methods

### 2.1 Study Area

**FIGURE 1:**
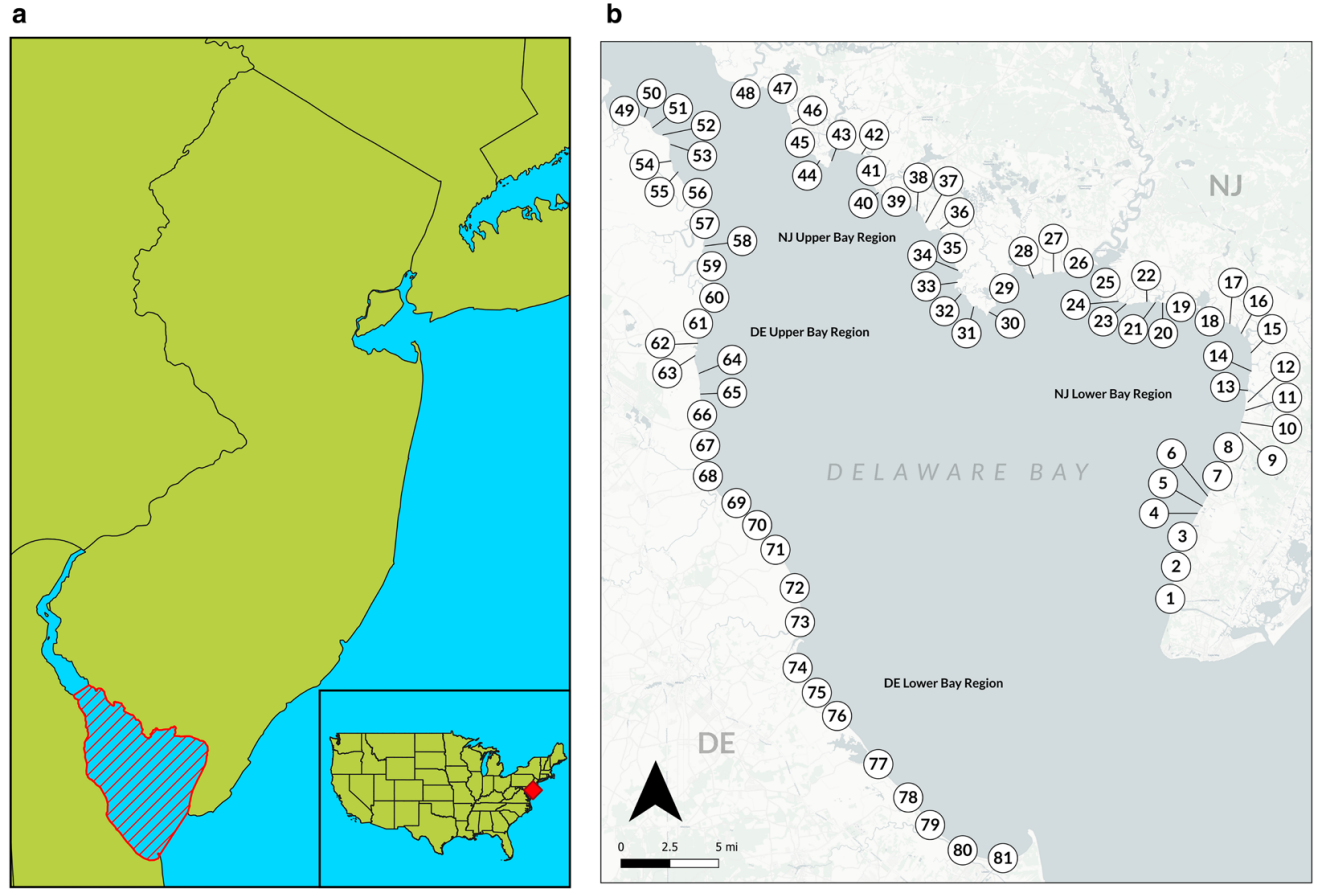
(a) Map of Delaware Bay region within New Jersey and the United States. (b) Map of 81 segment counts divided across four geographic regions: New Jersey (NJ) lower bay, NJ upper bay, Delaware (DE) upper bay, and DE lower bay. Surveys were conducted annually during the May spring stopover, 1986–2025. Inset: Location of Delaware Bay within the eastern United States. Each segment name is listed out under the bay regions and numbered; see Table S1 for all segment names.

Delaware Bay, a large estuarine system located along the mid‐Atlantic coast of the United States, represents one of the most important spring stopover sites for migratory shorebirds in the Western Hemisphere (Baker et al. 2004, Duijns et al. 2017, Niles et al. 2008; Figure 1). During their northbound migration, extensive intertidal beaches along the bay provide critical foraging habitat for shorebirds stopping over every May to refuel before continuing their migration north to their Arctic breeding grounds.

For this study, the Delaware Bay shoreline was divided into two opposing shorelines—the New Jersey (NJ) shore on the eastern side of the bay and the Delaware (DE) shore on the western side, separated by ~20 km of open water at the bay's widest point. The binary east–west partition was selected because the two shorelines have been managed independently across the time series (Smith et al. 2020), differ in geomorphology and beach restoration history, and represent the smallest spatial division at which a within‐bay redistribution signal could be reliably detected with peak‐count data. Each shoreline was further divided into a lower‐bay (mouth‐ward) and upper‐bay (river‐ward) sector, NJ lower bay, NJ upper bay, DE upper bay, and DE lower bay (Figure 1), but these four sectors are used here only as cartographic labels for organizing the 81 individual survey segments (Table S1) and are not treated as independent analytical units; we make no claim that they reflect how birds perceive or partition the bay. Though these shorelines differ in geomorphology, accessibility, as well as patterns of human use and management, together they encompass the habitat used by migratory shorebirds stopping over in the bay during May. Comparing peak counts between the two shorelines allowed us to document within‐bay redistribution while maintaining consistency across the multi‐decadal time series.

### 2.2 Peak Count Data

Annual peak counts (defined as the highest number of individuals of each species recorded on a single survey day) were obtained during the May migratory stopover from 1986 to 2025. Surveys were conducted by various grounds throughout the years including NJDEP, Wildlife Restoration Partnerships, Conserve Wildlife Foundation, DNREC, and other collaborating partners using a standardized aerial and ground count protocol (Clark et al. 1993; Niles et al. 2008). Aerial surveys formed the consistent backbone of the dataset from 1986 to 2025, supplemented by ground and boat‐based surveys beginning in 2013. Although survey methods and spatial coverage varied modestly among years due to logistical constraints and evolving protocols (Table 1), surveys were consistently conducted during peak migration to capture maximum shorebird abundance each year.

**TABLE 1:** Survey methodology used to estimate peak shorebird abundance at the Delaware Bay, USA, spring stopover, 1986–2025.

Year	No. of aerial surveys	No. of boat surveys	No. of ground surveys	Species counted	Comments
1986–2008	6	0	0	6	One counter, one apportitioner (decided % of flock a species was). Surveys once a week, no ground or boat counts. All spp. were counted REKN, RUTU, SAND, SBDO, DUNL, SESA.
2009–2015	2	2	2	2	2 counters, each counted one species (REKN & RUTU). Surveys conducted at peak stopover period May 18–30. Ground and boat count added and conducted with aerial surveys.
2016–2019	2	2	2	6	Surveyors changed. All 6 species counted, however, SBDO & DUNL were classified as medium peep, SESA classified as peep.
2020	0	2	2	4	COVID‐19 restricted the team from conducting aerial surveys. Only boat and ground counts were conducted. Species counted REKN, RUTU, SAND, SESA.
2021–2022	2	2	2	4	Protocol from 2015 to 2019 resumed. 2 Counters in the plane, one counted REKN & RUTU, the other counted SAND & SESA. Counts done during peak migration period. Boat and ground counts conducted at the same time.
2023‐current	2	2	2	4	Counts done during peak migration period. Boat and ground counts conducted at the same time. Aerial surveyors changed and only counted REKN & RUTU, ground/boat counted all four spp. (REKN, RUTU, SAND, SESA).

*Note:* The number of surveys per type per year, species counted, and survey protocols are summarized for each period. For the surveys conducted in Delaware Bay.

Abbreviations: DUNL, Dunlin; REKN, Red Knot; RUTU, Ruddy Turnstone; SAND, Sanderling; SBDO, Short‐billed Dowitcher; SESA, Semipalmated Sandpiper.

Aerial surveys in Delaware Bay were conducted annually, surveying about 80 km of beach in New Jersey and 80 km of beach in Delaware during the northbound stopover in May. All surveys were conducted in a Cessna high‐wing airplane, beginning on the New Jersey side of the bay at Town Bank (38°58′28.0″N 74°57′44.0″W) and ending on the Delaware side at Cape Henlopen (38°46′59.4″N 75°07′19.1″W) (Figure 1; Clark et al. 1993). Aerial surveys were divided into 81 segments; there are two survey counters on each survey and one person recording GPS locational data (Figure 1; Table S1). The plane flew at an altitude of approximately 30–60 m above ground level (100–200 ft) approximately 25–30 m seaward of the mean high tide line (i.e., over the water just off the beach face) at approximately 110 km/h, temporarily flushing birds off the beach. This allowed surveyors to count and identify birds in the air as the plane passed over. Surveys were conducted about 1 h after high tide, to coincide with peak shorebird concentrations on the beaches, when detectability was highest. Aerial surveys in the bay began in 1986 and targeted six shorebird species—Red Knots (REKN), Ruddy Turnstones (RUTU), Sanderling (SAND), Short‐billed Dowitcher (SBDO), Dunlin (DUNL), and Semipalmated Sandpipers (SESA) along the bay's shoreline beginning in the first week of May through the first week in June (Clark et al. 1993, Burger, Dey, and Niles 2024). Aerial surveys have been a standard method for monitoring large concentrations of migratory shorebirds at coastal stopover sites for decades (Howe et al. 1989; Frederick et al. 2003) and provide a reliable index of relative abundance when conducted with consistent protocols. Aerial counts at Delaware Bay underestimate the snapshot stopover population by approximately 17% relative to mark‐resight estimates (Lyons et al. 2015; Smith et al. 2022), but this bias is consistent across years given the stable protocol, preserving the comparability of trends over time.

In 2013 bay wide ground and boat counts were instituted to add more certainty to the aerial counts, especially in areas with high densities of birds (i.e., Mispillion Harbor, DE and Egg Island, NJ). Ground counts are conducted with one counter on a beach. The counter arrives 1 h before the plane and counts every 30 min continuing 1 h after the plan flies over. The count when the plane flies is recorded separately and compared with the aerial count. Boat counts are done with one surveyor, one boat captain and one recorder. As the boat travels slowly along the coast, the surveyor counts each species. In New Jersey two boat counts are used (covering different zones) in areas that are not accessible on foot (Table 1). With the ground and boat count methods, surveyors only use binoculars to count and identify birds. Whereas with the aerial surveys, surveyors count and identify birds from the air with their naked eye.

For each species and year, the annual peak count was defined as the highest number of individuals recorded on any single survey day during the stopover period (Table 2). Peak counts were used as a measure of relative abundance, helping to reduce bias from birds' short‐term movements and daily changes in their distribution.

**TABLE 2:** Annual peak counts (the highest number of individuals of each species recorded on any single survey day) for four shorebird species at the Delaware Bay, USA, spring stopover, 1986–2025, derived from aerial, ground, and boat‐based surveys.

Year	Red Knot	Turnstone	Sanderling	Semipalmated Sandpiper
1986	55,531	88,164	33,795	267,348
1987	38,750	69,448	28,472	91,790
1988	38,190	71,580	12,330	71,265
1989	94,460	105,160	8785	80,462
1990	45,785	32,301	7015	48,185
1991	27,280	42,020	3530	68,300
1992	25,595	53,930	7330	42,630
1993	44,000	64,985	10,390	91,080
1994	52,055	80,795	9955	95,180
1995	38,600	70,370	10,130	81,235
1996	19,445	47,115	8355	41,190
1997	41,855	69,340	15,455	75,395
1998	50,360	101,660	23,520	69,470
1999	49,805	87,640	10,005	84,755
2000	43,145	69,000	20,815	102,380
2001	36,125	86,365	21,830	192,205
2002	31,695	64,690	13,765	54,691
2003	16,255	34,975	11,560	38,615
2004	12,865	44,815	5135	77,325
2005	15,345	42,995	12,765	59,160
2006	13,445	18,585	16,740	93,785
2007	12,375	37,430	8870	75,779
2008	15,395	21,300	13,415	60,500
2009	13,361	17,135	—	—
2010	14,475	18,231	—	—
2011	12,804	13,440	—	—
2012	10,514	24,030	39	109,815
2013	17,377	17,659	4950	—
2014	24,980	22,161	—	2843
2015	24,890	21,360	5275	—
2016	21,128	16,153	9251	57,405
2017	17,969	17,488	3501	162,368
2018	32,930	41,895	20,369	97,530
2019	30,880	21,699	8440	93,185
2020	19,397	23,822	—	—
2021	6880	10,785	16,965	48,225
2022	12,114	11,222	25,510	68,087
2023	22,202	25,884	13,896	97,728
2024	14,225	21,481	13,951	100,492
2025	25,667	21,681	18,011	118,084

*Note:* Blank cells indicate years in which no count was completed for that species.

Since we did not have consistent count data for SBDO and DUNL past 2008, we did not include them in our analysis and used the peak count data available for four focal species: Red Knot (REKN), Ruddy Turnstone (RUTU), Sanderling (SAND), and Semipalmated Sandpiper (SESA). Data coverage across the time series varied among species, with some data gaps occurring in certain years, particularly for Sanderlings and Semipalmated Sandpipers (Table 1). Species‐specific analyses were therefore conducted using all years with available data.

#### 2.2.1 Data Limitations

Several caveats apply to interpretation of the long‐term peak count dataset. First, peak counts represent a relative index of stopover‐period abundance rather than absolute density, and detection probability was not formally estimated. The aerial survey protocol, however, has remained largely consistent in observer training, flight path, altitude, speed, and timing relative to peak migration since 1986 (Clark et al. 1993; Niles et al. 2008; Frederick et al. 2003), preserving comparability of relative abundance through time. In particular, although the number of counts per year varied (Table 1), all counts were conducted during the same peak migration window each May, and the maximum daily count was retained as the annual peak; this protocol consistency preserves the comparability of peak values across years even when survey effort differed. The strength of the long‐term decline signal for Red Knots and Ruddy Turnstones (*p* < 0.001) further indicates that the observed trends are robust to inter‐annual variation in survey effort. Second, the addition of ground and boat‐based counts beginning in 2013 was implemented to improve count accuracy in high‐density areas where aerial detection is most challenging (e.g., Mispillion Harbor, Egg Island) and to allow within‐year cross‐validation; for each year these surveys overlap in space and time with aerial counts, and the highest of the same‐day estimates is retained as the peak. Third, although coverage of Red Knots and Ruddy Turnstones has been consistent throughout the full time series, Sanderlings and Semipalmated Sandpipers were not consistently identified during aerial surveys in some intervals (Table 1), yielding gaps in their species‐specific records. We treat these features explicitly in interpretation rather than excluding affected years. Fourth, our among‐shoreline comparison treats the New Jersey and Delaware coasts as two units of comparison; we did not model habitat covariates (e.g., beach geomorphology, prey availability, disturbance intensity) within or among shorelines. The shoreline‐use patterns we describe should therefore be interpreted as documentation of where birds occur and how that distribution has shifted, rather than as a mechanistic spatial analysis of the drivers of redistribution.

### 2.3 Statistical Analyses

**FIGURE 2:**
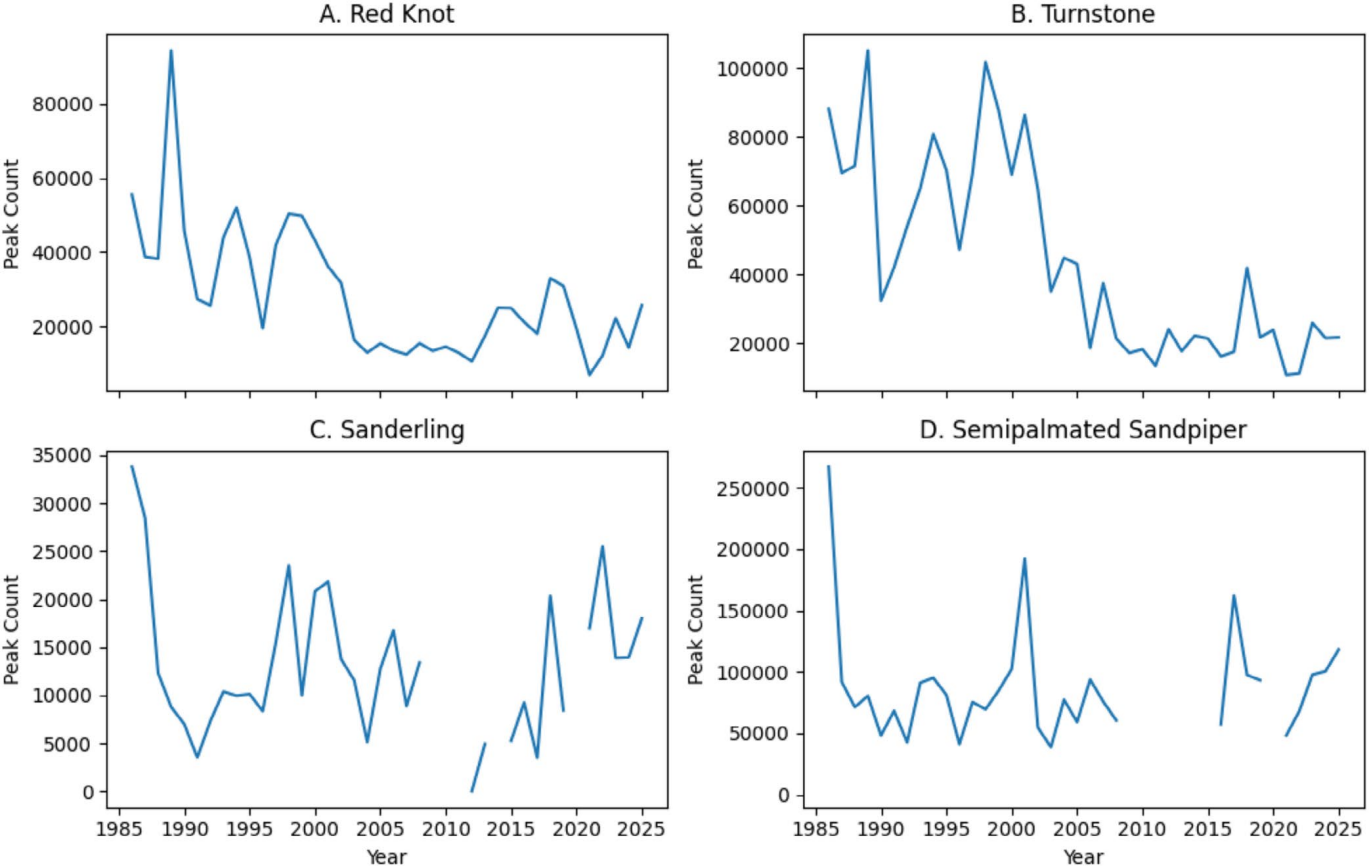
Annual peak count trends for four shorebird species during the May stopover in Delaware Bay, 1986–2025. Panels show (A) Red Knot, (B) Ruddy Turnstone, (C) Sanderling, and (D) Semipalmated Sandpiper. Peak counts represent the maximum number of individuals recorded during annual surveys. Missing values indicate years in which species‐specific peak counts were not collected for the corresponding species at Delaware Bay, USA.

Long‐term population trends were evaluated using log‐linear regression models, with annual peak counts as the response variable and year as a continuous predictor (Figure 2). Log transformation of peak counts allowed trends to be assessed in terms of proportional change and minimized the influence of years with exceptionally high counts. The statistical significance of yearly trends was assessed using hypothesis tests of regression coefficients, *p* ≤ 0.05. Species exhibiting statistically significant decreases in peak counts and consistent year‐to‐year data coverage across both shorelines were retained for further analysis of shoreline use; incomplete and inconsistent year‐to‐year spatial coverage for Sanderlings and Semipalmated Sandpipers precluded reliable estimation of among‐shoreline proportions for those species in many years (see Table 2 for the years lacking species‐specific data). Specifically, these two species were not consistently identified or recorded by aerial surveyors during several intervals (2009–2015 and 2020–2022; Table 2), producing large gaps in the shoreline‐level time series. For completeness, exploratory analyses of New Jersey shoreline proportions for Sanderlings and Semipalmated Sandpipers, restricted to years with complete data, are provided in Supporting Information (Appendix S2) but are not the focus of this study. Species without statistically significant declines along with consistent data coverage throughout the dataset were selected for further analysis of shoreline use (Figure 2, Table 3).

**TABLE 3:** Results of log‐linear regression analyses (log(peak count) ~ Year, fitted by ordinary least squares) evaluating long‐term trends in annual peak counts for four shorebird species at the Delaware Bay May stopover, 1986–2025.

Species	*n*	Slope (log scale)	SE	*t*	*p*	*R* ^2^	*F*	Interpretation
Red Knot	40	−0.031	0.006	−5.03	< 0.001	0.40	25.3	Significant decline (3.1%/year)
Ruddy Turnstone	40	−0.045	0.006	−7.98	< 0.001	0.63	63.7	Significant decline (4.4%/year)
Sanderling	35	−0.009	0.016	−0.55	0.59	0.01	0.30	Not significant
Semipalmated Sandpiper	34	−0.004	0.010	−0.42	0.68	0.01	0.18	Not significant

*Note:* No additional covariates were included. Slopes represent the per‐year change in log peak count; back‐transformed, this corresponds to a 3.1% per‐year decline in Red Knot peak counts and a 4.4% per‐year decline in Ruddy Turnstone peak counts. Years missing for Sanderling (*n* = 35) and Semipalmated Sandpiper (*n* = 34) reflect species with incomplete annual coverage (Table 2). Negative slopes indicate declining trends. Statistical significance was assessed at α = 0.05.

**FIGURE 3:**
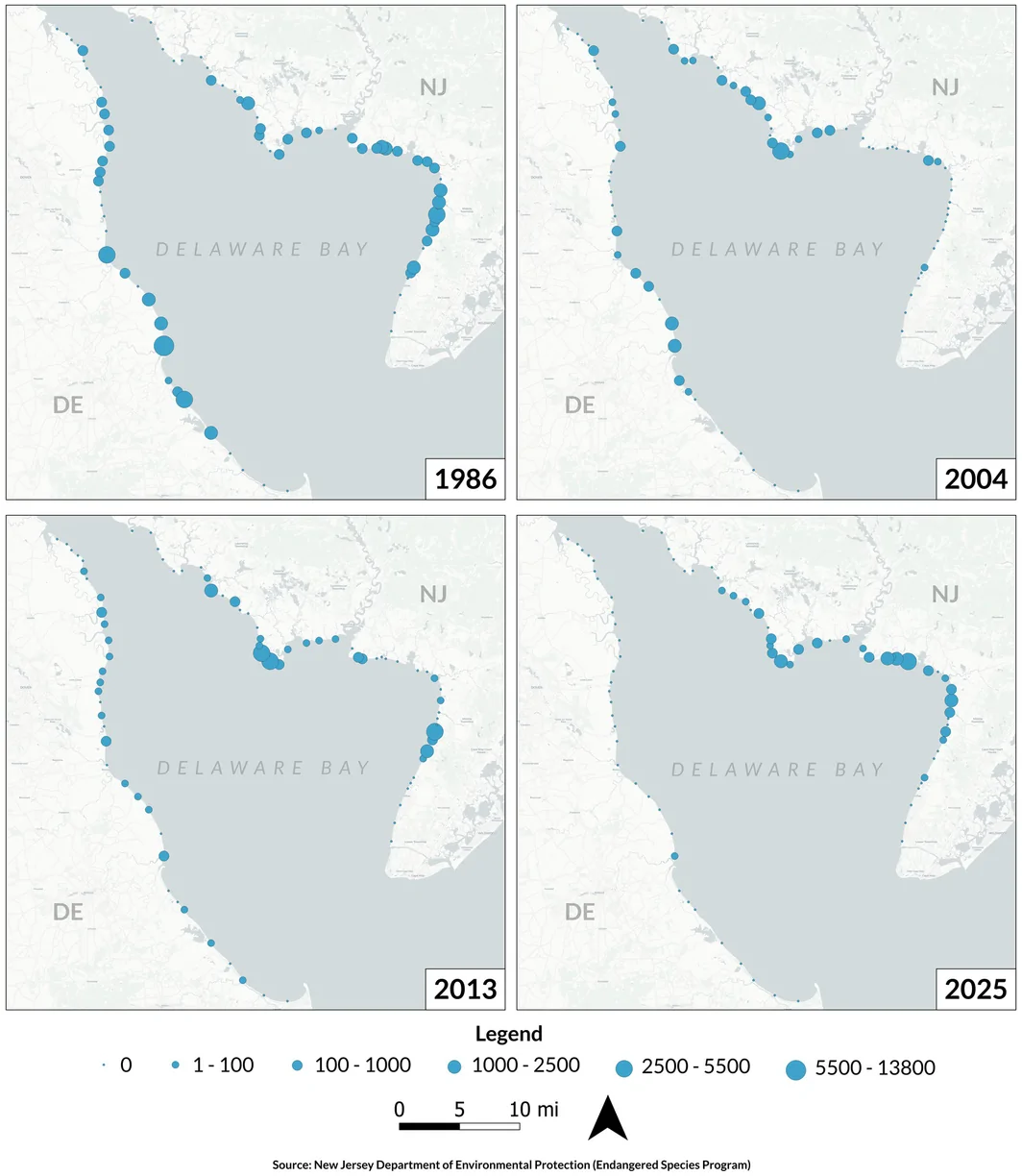
Example maps of aerial survey counts of red knots in Delaware Bay in 1986, 2004 after the population crash, 2013 after beach restoration work conducted in the bay, and 2025, showing a shift in distribution of red knots from bay wide use to favoring specific sites.

**FIGURE 4:**
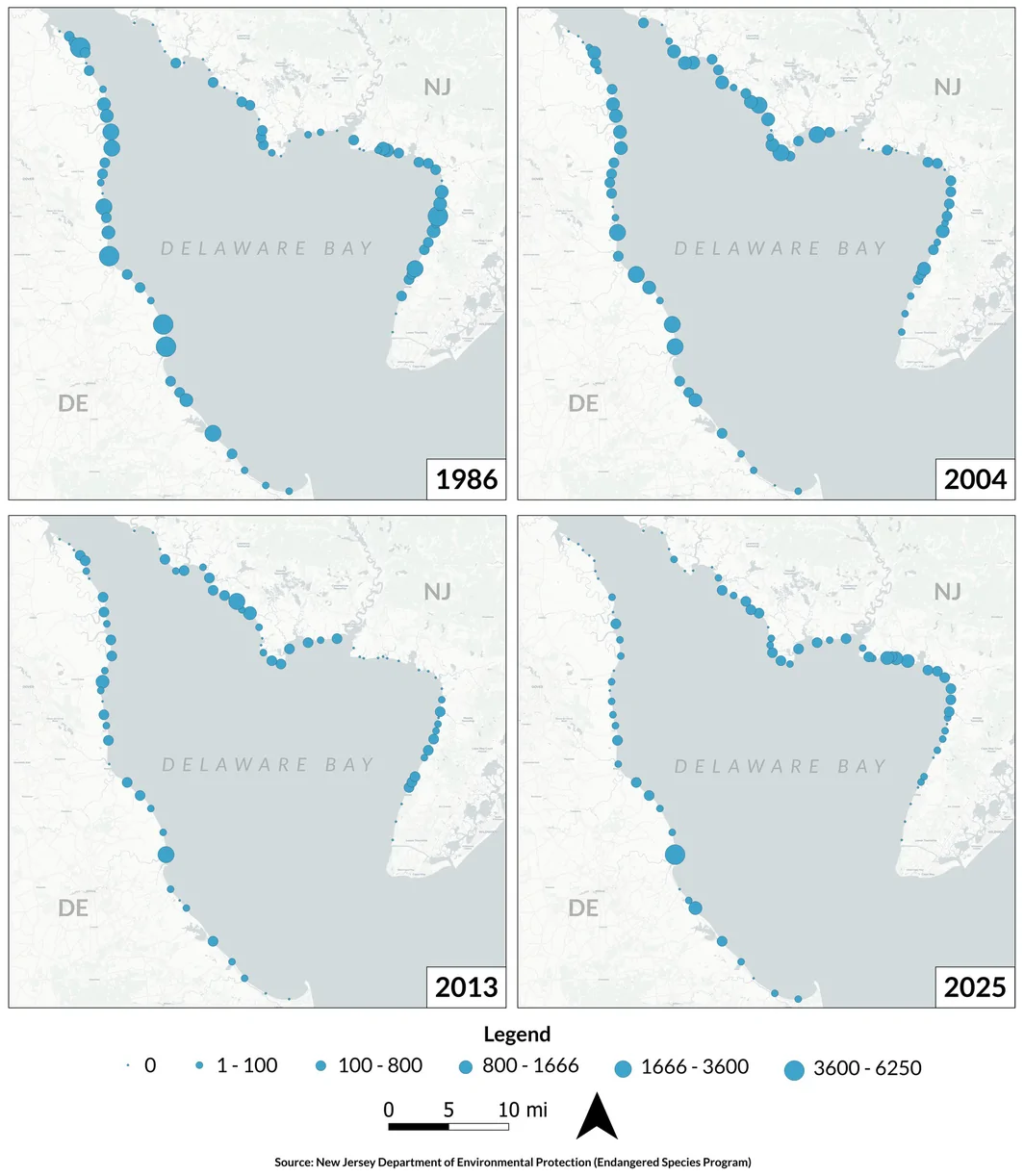
Example maps of aerial survey counts of Ruddy Turnstones in Delaware Bay in 1986, 2004 after the population crash, 2013 after beach restoration conducted in the bay, and 2025, showing a sustained, more even use of the bay over time.

**FIGURE 5:**
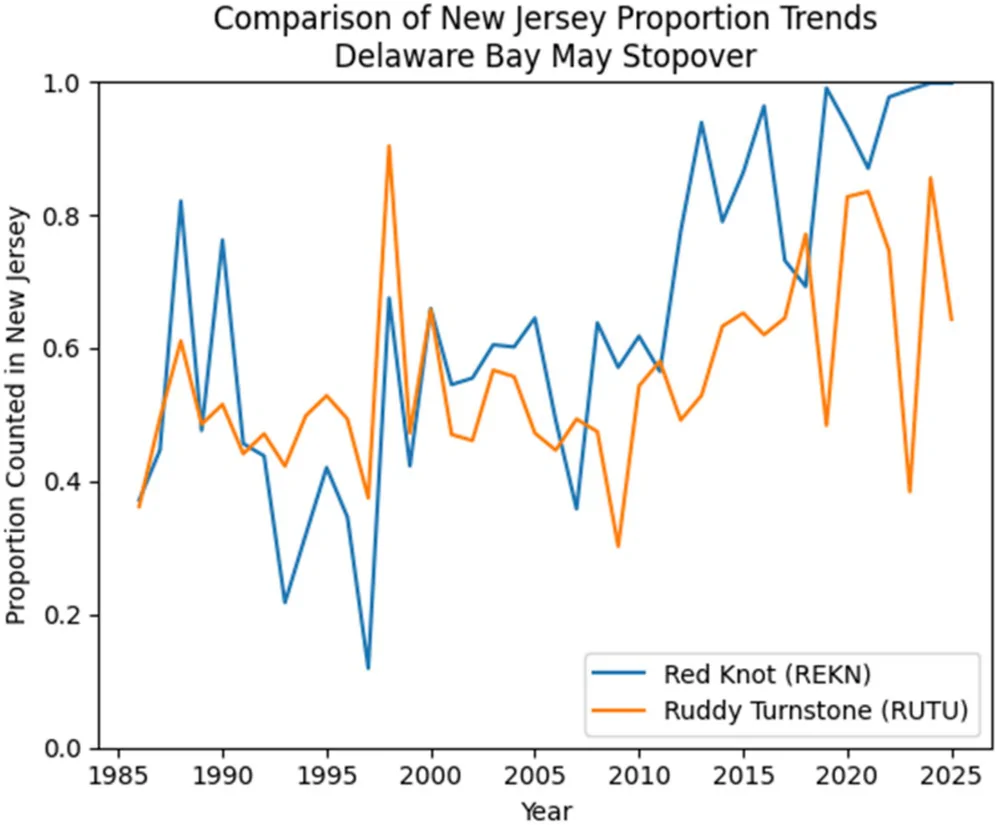
Temporal trends in the proportion of annual peak counts occurring on the New Jersey shoreline for Red Knots and Ruddy Turnstones during the Delaware Bay May stopover, 1986–2025.

Field observations from 2003 onward documented a spatial shift within the bay that accompanied the sharp decline in horseshoe crab egg densities after shorebird populations first began to fall (USFWS 2014a, 2014b). Temporal trends in shoreline use were then examined with log‐linear regression and interaction models to test species‐specific differences in shoreline‐use patterns year to year (Figures 3, 4, 5).

## 3 Results

### 3.1 Long‐Term Peak Count Trends Across Shorebird Species

Annual peak counts collected during the May stopover period from 1986 to 2025 showed both interannual variability with differences in long‐term trends across the four focal shorebird species (Figure 2, Table 3). General trends show year‐to‐year fluctuations in species peak counts with a drastic decline in each species in 2003. Peak counts for each species remained at lower levels until 2014 when a slight increase in counts can be seen for each species. However, all population counts remained at lower levels than historic numbers before their population crashes in 2003 (Table 2). While all four species show a decline in annual peak counts from the first count in 1986, both only REKN and RUTU annual counts showed statistically significant declines across the full dataset (Figure 2, Table 3).

Red Knot peak counts declined significantly over the study period, decreasing from 94,460 in the late 1980s to below 15,000 in the early 2000s, with partial increases observed in some recent years (Figure 2). Peak counts for Ruddy Turnstones followed a similar pattern of statistically significant declines long term, with higher counts in the late 1980s followed by sustained reductions and lower, more variable peak counts in subsequent decades (Figure 2).

In contrast, Semipalmated Sandpipers and Sanderling peak counts did not show the same consistent declines across the full‐time series. While both species counts show declines from historic numbers, the declines are not statistically significant (Figure 2). Sanderling counts were consistently lower compared to other species with high year‐to‐year variability, including periods of reduced detection and intermittent increased detection in recent years. Semipalmated Sandpipers consistently occurred at the highest abundances among the four species, often exceeding 90,000 individuals in peak years, but also showed year‐to‐year fluctuations in peak counts rather than a sustained long‐term decline throughout the dataset. Additionally, count data for both Sanderling and Semipalmated Sandpipers were not consistently collected throughout the full dataset (Tables 1 and 2).

Log‐linear regression analyses for each species showed a statistically significant decline in Red Knot and Ruddy Turnstone populations from 1986 to 2025 (*p* < 0.001), but no statistically significant decline for SESA and SAND (*p* > 0.5) populations across the full dataset (Figure 2, Table 3). While our regression analysis does not show a statistically significant decline for Semipalmated Sandpipers and Sanderling, a downward trend in population counts can be seen (Table 3). The *p* value results align with our figures showing statistically significant downward trends in two of the four species population estimates based on yearly peak counts (Table 3). Since statistically significant long‐term declines were detected only for Red Knot and Ruddy Turnstones, we focused further analysis on only these two species.

### 3.2 Shifts in Shoreline Use

Given the significant changes in peak counts for both Red Knot and Ruddy Turnstones, we decided to assess whether there were spatial shifts and if these shifts differed by species. Over time, Red Knots exhibited a pronounced and sustained shift in shoreline use from a relatively uniform distribution along both shorelines to a high concentration on the New Jersey side of the bay (Figure 3). During the late 1980s and early 1990s, Red Knot peak counts were more evenly distributed along both the New Jersey and Delaware shorelines, with New Jersey accounting for approximately 35%–75% of birds depending on the year (Figure 3). By the early 2010s, however, the proportion of Red Knots counted on the New Jersey side of the bay increased sharply and remained consistently high in subsequent years. From 2019 to 2025, more than 85% of all Red Knots counted in the bay resided on the New Jersey side of the bay, exceeding 97% in four of the most recent 5 years (Figure 5).

Ruddy Turnstones, in contrast, did not show the same distribution shift in shoreline use as Red Knots (Figure 4). The proportion of Ruddy Turnstones counted on the New Jersey shoreline varied year‐to‐year, with values fluctuating between 35% and 65% across the time series. Although Ruddy Turnstone use of the New Jersey side of the bay did increase in some years, this was not consistently sustained and the Delaware shoreline does still support many Ruddy Turnstones, even in recent years (Figure 5). Therefore, long‐term shoreline‐use patterns for Ruddy Turnstones do not seem to be characterized by a persistent spatial redistribution toward a single shoreline.

When comparing both species, formal analyses confirmed that temporal trends in shoreline use differed between Red Knots and Ruddy Turnstones (Figure 5). Figure 5 shows that while the proportion of Red Knots occurring on the New Jersey shoreline increased significantly over time, Ruddy Turnstones exhibited a substantially weaker and more variable pattern of shoreline use throughout the dataset (*p* = 0.001; Table 4).

**TABLE 4:** Results of linear interaction models evaluating species‐specific trends in the proportion of annual peak counts occurring on the New Jersey shoreline.

Term	Estimate	SE	*t*	*p*	Interpretation
Intercept (REKN at 2005.5)	0.641	0.023	28.28	< 0.001	Mean NJ proportion mid‐series
Year (REKN slope)	+0.015	0.002	7.67	< 0.001	Increasing NJ shoreline use
Species (RUTU vs. REKN)	−0.086	0.032	−2.68	0.009	Lower NJ use by RUTU at mid‐series
Year × Species	−0.009	0.003	−3.39	0.001	Weaker temporal trend for RUTU

*Note:* Full model formula and fit statistics are reported in the table footer. Positive slopes indicate increasing proportion of peak counts occurring on the New Jersey shoreline. Model: proportion_NJ ~ Year × Species, fitted by ordinary least squares. *n* = 80 species × year observations (Red Knot *n* = 40, Ruddy Turnstone *n* = 40). Reference species: Red Knot. Year is centered on 2005.5 (midpoint of the 1986–2025 time series). Model fit: *R* ^2^ = 0.49, adjusted *R* ^2^ = 0.47, *F*(3, 76) = 24.80, *p* < 0.001; residual SE = 0.144; AIC = −79.7. No additional covariates were included.

## 4 Discussion

Using nearly four decades of standardized peak count data, this study documents long‐term population changes and spatial redistribution of migratory shorebirds at the Delaware Bay spring stopover. In the discussion that follows, we use “shorebirds” when referring broadly to the four focal species and the Delaware Bay stopover community, and species names when referring to species‐specific findings. The redistribution toward the New Jersey shoreline documented here is specific to Red Knots; Ruddy Turnstones declined in peak counts but did not show consistent redistribution, and shoreline use was not analyzed for Sanderlings or Semipalmated Sandpipers. Although all four focal species exhibited substantial interannual variability in peak counts, only Red Knots and Ruddy Turnstones showed statistically significant long‐term declines. This is in keeping with global trends reported in Smith et al. 2023 for the period from 1980 to 2019, where Red Knots have reportedly declined in the Western Hemisphere by about 98%, Ruddy Turnstones have declined by about 75%, Semipalmated Sandpipers have declined by about 68%, and Sanderlings have declined by about 45%. Much of the attention to date has focused on Red Knots since the initial population crash and as they have suffered the greatest population declines out of the 28 shorebird species surveyed throughout the hemisphere (Niles et al. 2008; Baker et al. 2013; Duijns et al. 2017; Burger, Dey, and Niles 2024; Smith et al. 2023). Though the four species included in our analysis exhibit variation in their wintering grounds, breeding grounds, and migratory routes, all four are among the shorebird species with the steepest documented declines in the Western Hemisphere (Baker et al. 2004; Niles et al. 2008; Smith et al. 2023) at the same time. The trends we observed at Delaware Bay are consistent with these flyway‐wide declines for Red Knots and Ruddy Turnstones, although declines for Sanderlings and Semipalmated Sandpipers in our dataset were not statistically significant. Long‐standing concerns about the Delaware Bay stopover's degraded condition (Baker et al. 2004, Burger et al. 2017, Burger, Dey, and Niles 2024, Smith et al. 2020, 2022). This emphasizes the importance of protecting this site not only for bird species.

### 4.1 Shifts in Shoreline Use

A central finding of this study is the pronounced spatial redistribution of Red Knots within Delaware Bay. Anecdotally, since the decline of shorebird populations in 2003 due to the major decline of horseshoe crab egg densities, a spatial use shift within the bay has been noted for Red Knots (USFWS 2014a, 2014b, 2016; Burger, Dey, and Niles 2024; Smith et al. 2020). These field observations have documented a substantial shift in the distribution of Red Knots throughout the bay, with specific areas being favored throughout the season (consistent with field observations summarized in Burger, Dey, and Niles 2024) throughout the season. Our analysis shows that over time, Red Knot shoreline use has shifted from a relatively even distribution along both shorelines to a high concentration along the New Jersey shoreline, with almost 99% of the bay's population recorded there in recent years. In contrast, Ruddy Turnstones, despite experiencing a statistically significant decline in peak counts, continued to use both the New Jersey and Delaware shorelines throughout the study period. This divergence suggests that the pronounced spatial redistribution observed in Red Knots reflects species‐specific sensitivity to within bay conditions rather than a bay‐wide shift affecting all shorebirds.

### 4.2 Red Knot Distribution Shift

One plausible driver of the redistribution observed in Red Knots is variation in human disturbance between the two shorelines. All four focal species depend on horseshoe crab eggs during stopover (Tsipoura and Burger 1999), but Red Knots may be especially constrained by the brief window during which they must achieve large mass gains for non‐stop migration to the Arctic (Baker et al. 2004; Duijns et al. 2017). Red Knots are highly sensitive to disturbance during foraging, and high levels of disturbance can impact their ability to forage throughout the tide cycle during their short stopover period (Burger et al. 2004, 2007, 2013). After the decline in 2003, beach protection measures were put into place on the NJ beaches in Delaware Bay to protect foraging shorebirds from human disturbance. These 12 beaches in NJ are annually protected with temporary fencing roping off restricted areas, informative signage explaining the migration and closure, as well as stewards at some beaches to enforce signage and provide information to the public. This protection from disturbance could be a reason for the shift in beach use (Burger, Dey, and Niles 2024; Dey et al. 2021).

A second plausible driver is the quality and availability of horseshoe crab spawning habitat, which directly determines the surface egg supply available to foraging shorebirds (Smith et al. 2020, 2022). The large‐scale restoration effort on New Jersey Delaware Bay beaches from 2012 onward provides one of the clearest tests of this relationship. After superstorm Sandy hit Delaware Bay in 2012 nearly 70% of horseshoe crab habitat on the New Jersey side of the bay was lost, which would have had a large negative impact on shorebirds and horseshoe crabs that following May. In response, a beach restoration project was launched in New Jersey to restore over a mile of beach before the spring horseshoe crab breeding season (Niles et al. 2013; Smith et al. 2020). One of the main reasons this project was a success was due to the design of the restored beaches. The beaches were designed to support horseshoe crab breeding, with profiles tailored to site ecology rather than primarily to human recreation, as is common in some restoration projects. In addition, each beach was restored with a sand grain size of 0.6–0.9 mm, which is optimal for horseshoe crab breeding (Smith et al. 2022). This grain size helps maintain the oxygenation needed for egg survival (Smith et al. 2020, 2022).

Restoration projects have continued on the New Jersey side, with efforts continuing to focus on expanding high‐quality horseshoe crab breeding habitat and increasing surface egg availability for shorebirds during stopover (Smith et al. 2020, 2022). Importantly, the patterns described above represent a shift in where Red Knots are distributed within the bay rather than a recovery in total numbers; bay‐wide peak counts have remained well below historic levels throughout the time series (Figure 2). While restoration has occurred on the Delaware side of the bay, those projects have not necessarily followed restoration design plans focused on increased surface egg availability and horseshoe crab breeding, potentially making them less suitable. Previous research has shown that Red Knots are highly dependent on localized foraging conditions during stopover and are sensitive to variation in habitat quality and prey availability (Burger et al. 2004, 2007, 2013a). Changes in habitat quality, prey distribution, or disturbance regimes within the bay may therefore influence shoreline selection (Smith et al. 2020, 2022). However, because this study did not directly evaluate prey availability, disturbance intensity, or habitat quality, these factors remain hypothesized drivers of redistribution. Explicit integration of spatially resolved prey and disturbance data with long‐term count data will be necessary to test these mechanisms.

Differences in feeding ecology and disturbance tolerance between Red Knots and Ruddy Turnstones likely explain why these two declining species showed such different patterns of within‐bay shoreline use. Red Knots primarily forage on surface eggs, with feeding success tied closely to the availability of horseshoe crab eggs on the surface of the beach (Tsipoura and Burger 1999; Niles et al. 2008). Additionally, Red Knots are among the most disturbance‐sensitive shorebirds at the bay, with even brief human or vehicle activity reducing the time available for foraging during the short stopover window (Burger et al. 2004, 2007, 2013a). Ruddy Turnstones, in contrast, can probe and dig to access buried egg clusters when surface eggs are unavailable, and are considerably less reactive to human disturbance (Tsipoura and Burger 1999, Burger et al. 2013a). Among the four species considered here, Red Knots therefore stand out as both the most reliant on surface egg availability and the most sensitive to disturbance, the two factors expected to differ most between the New Jersey and Delaware shorelines. Ruddy Turnstones, with their broader foraging repertoire and lower disturbance sensitivity, should respond less strongly to such within‐bay differences, consistent with the muted shoreline‐use trend we observed.

The sustained population reduction and increasing shoreline concentration documented here raise the question of whether Delaware Bay still functions as a high‐quality stopover or has shifted to operating as an ecological sink. Available evidence does not support a sink interpretation. Although shorebird populations remain at a fraction of historic numbers, the bay still retains key geomorphic features, latitudinal position, and biological infrastructure (an intact, though depleted, horseshoe crab spawning population) that historically supported orders‐of‐magnitude greater shorebird abundance (Clark et al. 1993; Niles et al. 2008; Smith et al. 2022). Targeted beach restoration projects on the New Jersey side have demonstrated that horseshoe crab egg densities can match or exceed reference beaches within a single spawning season when habitat is properly designed (Smith et al. 2020). The most likely reason shorebird populations have not recovered is not that the bay has become unsuitable, but that horseshoe crab populations, egg densities, and high‐quality breeding habitat remain suppressed below historic levels by ongoing harvest pressure and legacy shoreline degradation (Smith et al. 2017, Vision 2016, Smith et al. 2022). Recovery of the shorebird populations that depend on Delaware Bay, particularly Red Knots and Ruddy Turnstones, cannot occur while the bay itself remains held below its carrying capacity in both suitable foraging space and sufficient surface eggs. Protecting and restoring the resources the bay provides offers the most direct path to shorebird recovery.

In summary, this study demonstrates that long‐term peak count data, when combined with analyses of shoreline use, provide valuable insight into population dynamics and habitat use at a critical migratory stopover. In a time with shorebird population levels at about one quarter of historic numbers, these birds can be more selective in foraging and roosting sites, potentially selecting for sites with improved foraging conditions (e.g., greater surface egg availability) or reduced human disturbance. From a conservation perspective, these results highlight the critical role of long‐term, spatially explicit monitoring programs for continued understanding of shorebird population health over time. Therefore, continued conservation and management projects to help protect this important stopover and improve foraging conditions for shorebirds will be key to their recovery.

## Author Contributions

**Stephanie Feigin:** conceptualization (lead), data curation (lead), formal analysis (lead), funding acquisition (equal), investigation (lead), methodology (lead), writing – original draft (lead), writing – review and editing (lead). **Joanna Burger:** conceptualization (equal), supervision (equal), writing – review and editing (equal). **Theo Diehl:** formal analysis (equal), software (equal), writing – review and editing (equal). **Lawrence Niles:** conceptualization (equal), resources (equal), supervision (equal), writing – review and editing (equal). **Amanda Dey:** data curation (supporting), funding acquisition (equal), resources (equal), writing – review and editing (equal).

## Funding

Over the years this project has been funded by New Jersey Natural Lands Trust, U.S. Fish and Wildlife Service Office of Conservation Investment, NJDEP Fish and Wildlife, Delaware DNREC Division of Fish and Wildlife, and the National Fish and Wildlife Foundation.

## Ethics Statement

Ethics guidelines for Rutgers University followed for the development of this manuscript.

## Conflicts of Interest

The authors declare no conflicts of interest.

## Supporting information


**Appendix S1:** Data 1986–1996.

**Appendix S2:** Exploratory shoreline‐use analyses for Sanderlings and Semipalmated Sandpipers, Delaware Bay 1986–1996.

**Table S1:** Aerial survey segment names for shorebird peak count surveys at Delaware Bay, USA, during the May spring stopover, 1986–2025. The 81 segments are divided across four geographic regions (Figure 1). This table is paired with Figure 1.

## Data Availability

The annual peak count data (1986–2025) and the New Jersey and Delaware shoreline‐use data for Red Knots and Ruddy Turnstones that support the findings of this study are archived in the Dryad Digital Repository at [https://doi.org/10.5061/dryad.cc2fqz6nn]. Individual survey session data are available from the corresponding author upon reasonable request.

## References

[ece374183-bib-0001] Andres, B. A. , P. A. Smith , R. I. G. Morrison , C. L. Gratto‐Trevor , S. C. Brown , and C. A. Friis . 2013. “Population Estimates of North American Shorebirds, 2012.” Wader Study Group Bulletin 119: 178–194.

[ece374183-bib-0002] Atlantic States Marine Fisheries Commission . 1998. Interstate Fishery Management Plan for Horseshoe Crab. ASMFC.

[ece374183-bib-0003] Atlantic States Marine Fisheries Commission . 2009. “A Framework for Adaptive Management of Horseshoe Crab Harvest in the Delaware Bay Constrained by Red Knot Conservation.” November 2009, 46 p.

[ece374183-bib-0004] Baker, A. , P. Gonzalez , R. I. G. Morrison , and B. A. Harrington . 2013. “Red Knot ( *Calidris canutus* ).” In The Birds of North America Online, edited by A. Poole . Cornell Lab of Ornithology. https://birdsoftheworld.org/bow/species/redkno/cur/introduction.

[ece374183-bib-0005] Baker, A. J. , P. M. Gonzalez , T. Piersma , et al. 2004. “Rapid Population Decline in Red Knots: Fitness Consequences of Decreased Refueling Rates and Late Arrival in Delaware Bay.” Proceedings of the Royal Society of London. Series B: Biological Sciences 271, no. 1541: 875–882. 10.1098/rspb.2003.2663 PMC1691665 15255108

[ece374183-bib-0006] Botton, M. L. , R. E. Loveland , and T. R. Jacobsen . 1994. “Site Selection by Migratory Shorebirds in Delaware Bay, and Its Relationship to Beach Characteristics and Abundance of Horseshoe Crab ( *Limulus polyphemus* ) Eggs.” Auk 111: 605–616.

[ece374183-bib-0007] Burger, J. 1986. “The Effect of Human Activity on Shorebirds in Two Coastal Bays in Northeastern United States.” Environmental Conservation 13: 123–130.

[ece374183-bib-0008] Burger, J. , S. A. Carlucci , C. W. Jeitner , and L. Niles . 2007. “Habitat Choice, Disturbance, and Management of Foraging Shorebirds and Gulls at a Migratory Stopover.” Journal of Coastal Research 23: 1159–1166.

[ece374183-bib-0009] Burger, J. , A. Dey , and L. Niles . 2024. “Importance of Stewards and Other Protection Efforts for Conservation of Red Knots ( *Calidris canutus rufa* ) and Other Shorebirds on Delaware Bay, New Jersey.” Natural Science 16, no. 9: 162–182.

[ece374183-bib-0035] Burger, J. , and M. Gochfeld . 2016. Habitat, Population Dynamics, and Metal Levels in Colonial Waterbirds: A Food Chain Approach. CRC Press.

[ece374183-bib-0010] Burger, J. , C. Jeitner , K. Clark , and L. Niles . 2004. “The Effect of Human Activities on Migrant Shorebirds: Successful Adaptive Management.” Environmental Conservation 31: 283–288.

[ece374183-bib-0011] Burger, J. , and L. Niles . 2013. “Shorebirds and Stakeholders: Effects of Beach Closure and Human Activities on Shorebirds at a New Jersey Coastal Beach.” Urban Ecosystems 16, no. 3: 657–673.

[ece374183-bib-0012] Burger, J. , R. R. Porter , and L. J. Niles . 2024. “The Migratory Strategies of the Red Knots Using the Western Atlantic Flyway as Revealed by Geolocators.” Wader Study 131, no. 2: 93–105.

[ece374183-bib-0040] Burger, J. , N. Tsipoura , and M. Gochfeld . 2017. “Metal Levels in Blood of Three Species of Shorebirds During Stopover on Delaware Bay Reflect Levels in Their Food.” Horseshoe Crab eggs. Toxics 5, no. 3: 20. 29051452 10.3390/toxics5030020 PMC5634703

[ece374183-bib-0013] Clark, K. , L. J. Niles , and J. Burger . 1993. “Abundance and Distribution of Migrant Shorebirds in Delaware Bay.” Condor 95: 694–705.

[ece374183-bib-0014] Conklin, J. R. , P. F. Battley , M. A. Potter , and J. Fox . 2010. “Breeding Latitude Drives Individual Schedules in a Trans‐Hemispheric Migrant Bird.” Nature Communications 1: 67. 10.1038/ncomms1072 20842198

[ece374183-bib-0015] Dey, A. , L. J. Niles , S. Feigin , T. Diehl , and M. Pellew . 2021. Update to the Status of the Red Knot ( *Calidris canutus rufa* ), February 2021. Department of Environmental Protection.

[ece374183-bib-0016] Duijns, S. , L. J. Niles , A. Dey , et al. 2017. “Body Condition Explains Migratory Performance of a Long‐Distance Migrant.” Proceedings. Biological Sciences 284: 20171374. 29093218 10.1098/rspb.2017.1374 PMC5698639

[ece374183-bib-0017] Frederick, P. C. , B. Hylton , J. A. Heath , and M. Ruane . 2003. “Accuracy and Variation in Estimates of Large Numbers of Birds by Individual Observers Using an Aerial Survey Simulator.” Journal of Field Ornithology 74, no. 3: 281–287.

[ece374183-bib-0018] Galbraith, H. , D. W. DesRochers , S. Brown , and J. M. Reed . 2014. “Predicting Vulnerabilities of North American Shorebirds to Climate Change.” PloS One 9: e108899. 25268907 10.1371/journal.pone.0108899 PMC4182597

[ece374183-bib-0019] Galbraith, H. , R. Jones , R. Park , et al. 2002. “Global Climate Change and Sea Level Rise: Potential Losses of Intertidal Habitat for Shorebirds.” Waterbirds 25: 173–183.

[ece374183-bib-0034] Goss‐Custard, J. D. , A. D. West , M. G. Yates , et al. 2006. “Intake Rates and the Functional Response in Shorebirds (Charadriiformes) Eating Macro‐Invertebrates.” Biological Reviews 81, no. 4: 501–529. 16863594 10.1017/S1464793106007093

[ece374183-bib-0020] Howe, M. A. , P. H. Geissler , and B. A. Harrington . 1989. “Population Trends of North American Shorebirds Based on the International Shorebird Survey.” Biological Conservation 49: 185–199.

[ece374183-bib-0021] Lyons, J. E. , W. L. Kendall , J. A. Royle , S. J. Converse , B. A. Andres , and J. B. Buchanan . 2015. “Population Size and Stopover Duration Estimation Using Mark‐Resight Data and Bayesian Analysis of a Superpopulation Model.” Biometrics 71: 1–24. 26348116 10.1111/biom.12393

[ece374183-bib-0022] Marra, P. P. , E. B. Cohen , S. R. Loss , J. E. Rutter , and C. M. Onra . 2015. “A Call for Full Annual Cycle Research in Animal Ecology.” Biology Letters 11: 20150552. 26246337 10.1098/rsbl.2015.0552 PMC4571685

[ece374183-bib-0023] McGowan, C. P. , J. E. Hines , J. D. Nichols , et al. 2011. “Demographic Consequences of Migratory Stopover: Linking Red Knot Survival to Horseshoe Crab Spawning Abundance.” Ecosphere 2: 1–21.

[ece374183-bib-0024] Morrison, R. I. G. , Y. Aubrey , R. W. Butler , et al. 2001. “Declines in North American Shorebird Populations.” Wader Study Group Bulletin 94: 37–42.

[ece374183-bib-0025] Niles, L. J. , H. P. Sitters , A. D. Dey , et al. 2008. “Status of the Red Knot, *Calidris canutus rufa* , in the Western Hemisphere.” Studies in Avian Biology 36: 1–185.

[ece374183-bib-0026] Niles, L. J. , J. Smith , D. Daly , et al. 2013. “Restoration of Horseshoe Crab and Migratory Shorebird Habitat On Five Delaware Bay Beaches Damaged By Superstorm Sandy.” Published Electronically [Internet].

[ece374183-bib-0036] Senner, S. E. , and M. A. Howe . 1984. “Conservation of Nearctic Shorebirds.” In Shorebirds: Breeding Behavior and Populations, 379–421. Springer US.

[ece374183-bib-0027] Smith, J. A. , A. Dey , K. Williams , T. Diehl , S. Feigin , and L. J. Niles . 2022. “Horseshoe Crab Egg Availability for Shorebirds in Delaware Bay: Dramatic Reduction After Unregulated Horseshoe Crab Harvest and Limited Recovery After 20 Years of Management.” Aquatic Conservation: Marine and Freshwater Ecosystems 32, no. 12: 1913–1925.

[ece374183-bib-0038] Smith, J. A. , S. F. Hafner , and L. J. Niles . 2017. “The Impact of Past Management Practices on Tidal Marsh Resilience to sea Level Rise in the Delaware Estuary.” Ocean and Coastal Management 149: 33–41.

[ece374183-bib-0028] Smith, J. A. , L. J. Niles , S. Hafner , A. Modjeski , and T. Dillingham . 2020. “Beach Restoration Improves Habitat Quality for American Horseshoe Crabs and Shorebirds in the Delaware Bay, USA.” Marine Ecology Progress Series 645: 91–107.

[ece374183-bib-0029] Smith, P. A. , A. C. Smith , B. Andres , et al. 2023. “Accelerating Declines of North America's Shorebirds Signal the Need for Urgent Conservation Action.” Ornithological Applications 125: duad003.

[ece374183-bib-0030] Tsipoura, N. , and J. Burger . 1999. “Shorebird Diet During Spring Migration Stop‐Over on Delaware Bay.” Condor 101: 635–644.

[ece374183-bib-0031] U.S. Fish and Wildlife Service (USFWS) . 2014a. “Threatened Species Status for the Rufa Red Knot.” 79 Federal Register 238 (2014 December 11): 73706–73748.

[ece374183-bib-0032] U.S. Fish and Wildlife Service (USFWS) . 2014b. Rufa Red Knot Background Information and Threat Assessment. Supplement to Endangered and Threatened Wildlife and Plants; Final Threatened Status for the Rufa red knot ( *Calidris canutus rufa* ). [Docket No. FWS‐R5‐ES‐2013‐0097; RIN AY17], edited by U.S. Fish and Wildlife Service.

[ece374183-bib-0033] U.S. Fish and Wildlife Service (USFWS) . 2016. “Biological Opinion on the Effects of Existing and Expanded Structural Aquaculture of Native Bivalves in Delaware Bay, Middle and Lower Townships, Cape May County, New Jersey, on the Federally Listed Red Knot ( *Calidris canutus rufa* ).” April 2016. 175 p.

[ece374183-bib-0037] Vision, A. S. M. F. C. 2016. “Atlantic States Marine Fisheries Commission.” Management.

