# Supplementary material for: Long‐Term Population Trends and Spatial Redistribution of Shorebirds at a Critical Migratory Stopover
Source: Ecol Evol. 2026 Sep 2;16(9):e74183. doi: 10.1002/ece3.74183 (PMC13539063; doi:10.1002/ece3.74183)
Supplement: Supplementary file 1 [file ECE3-16-e74183-s001.docx]

**APPENDIX S2**

**Exploratory shoreline-use analyses for Sanderlings and Semipalmated Sandpipers, Delaware Bay 1986–1996**

*Supplementary material for: Feigin, S., Burger, J., Diehl, T., Niles, L., & Dey, A. Long-term population trends and spatial redistribution of shorebirds at a critical migratory stopover. Ecology and Evolution (MS ECE-2026-03-00710).*

**Purpose**

This appendix provides the exploratory shoreline-use analyses for Sanderlings (Calidris alba) and Semipalmated Sandpipers (Calidris pusilla) referenced in the main text. The analyses reported in the main manuscript (Table 5, Figure 5) are restricted to Red Knots and Ruddy Turnstones because these two species were the only ones with consistent, species-specific shoreline-level data across the full 1986–2025 time series. Here, we describe the temporal coverage limitations that prevented a full time-series shoreline-use analysis for Sanderlings and Semipalmated Sandpipers, and we present a descriptive analysis restricted to the subset of years in which shoreline-level counts for both species are complete.

**Data limitations**

Species-specific shoreline-level counts for both Sanderlings and Semipalmated Sandpipers are complete only in the 1986–1996 aerial survey period. From 1997 onward, small sandpipers were periodically pooled into aggregate categories on the datasheet, which compromised species-specific totals; from 2016 onward, aggregate categorization also affected Semipalmated Sandpipers directly. The result is a clean-data window that ends in 1996 and cannot be extended without introducing an unknown magnitude of species-level bias.

Aerial survey observers recorded shorebirds to species when field conditions allowed. When flock composition, distance, or lighting prevented reliable species-level identification, small sandpipers were pooled into aggregate categories on the datasheet ("PEEP" for small peeps, "MED/PEEP" for medium and small peeps combined). These aggregate categories included unknown proportions of Sanderling and Semipalmated Sandpiper, and in some years also included Short Billed Dowitcher (Limnodromus griseus) and Dunlin (Calidris alpina).

For 23 of the 40 years in the full time series (1997–2019), one or more surveys included non-zero counts in the PEEP or MED/PEEP categories, meaning that species-specific Sanderling and Semipalmated Sandpiper counts for those years underestimate their true abundance by an unknown amount. In a further six years (2009–2011, 2013–2015), aerial surveys were reduced in scope or interrupted, and shoreline-level totals for Sanderling and Semipalmated Sandpiper cannot be reconstructed reliably. These coverage limitations preclude any statistical assessment of long-term shoreline-use trends for these two species comparable to that reported for Red Knots and Ruddy Turnstones in the main text.

The only period with clean, species-specific, shoreline-level counts for both Sanderlings and Semipalmated Sandpipers is 1986–1996 (n = 11 years). During this window, aerial observers identified all small Calidris to species and PEEP and MED/PEEP counts were zero throughout. The exploratory analyses below are restricted to this window.

**Methods**

For each year 1986–1996, we identified the peak survey day for Sanderling and Semipalmated Sandpiper independently, defined as the survey with the greatest total (New Jersey + Delaware) count for that species. We then computed the New Jersey shoreline proportion as (NJ count) / (NJ count + DE count) on the peak day. Following the analytical approach used for Red Knots and Ruddy Turnstones in the main text, we tested for a temporal trend in the New Jersey shoreline proportion using simple linear regression of proportion on year. For direct comparison, we also refit the shoreline-use regression for Red Knots and Ruddy Turnstones over the same 1986–1996 window.

**Table S2.**

| **Year** | **SAND NJ** | **SAND DE** | **SAND prop. NJ** | **SESA NJ** | **SESA DE** | **SESA prop. NJ** |
| --- | --- | --- | --- | --- | --- | --- |
| 1986 | 14,130 | 19,665 | 0.418 | 100,337 | 167,011 | 0.375 |
| 1987 | 19,162 | 9,310 | 0.673 | 25,720 | 66,070 | 0.280 |
| 1988 | 12,105 | 225 | 0.982 | 99,329 | 103,690 | 0.489 |
| 1989 | 8,055 | 730 | 0.917 | 40,477 | 39,985 | 0.503 |
| 1990 | 6,350 | 665 | 0.905 | 24,700 | 23,485 | 0.513 |
| 1991 | 5,305 | 0 | 1.000 | 21,800 | 46,500 | 0.319 |
| 1992 | 7,210 | 120 | 0.984 | 14,485 | 28,145 | 0.340 |
| 1993 | 9,620 | 770 | 0.926 | 52,465 | 38,615 | 0.576 |
| 1994 | 7,220 | 2,735 | 0.725 | 39,720 | 55,460 | 0.417 |
| 1995 | 9,920 | 210 | 0.979 | 33,095 | 48,140 | 0.407 |
| 1996 | 4,765 | 3,590 | 0.570 | 26,325 | 14,865 | 0.639 |
| **Mean** | **9,440** | **3,456** | **0.825** | **43,496** | **57,997** | **0.442** |

**Table S2:** Clean-year shoreline-level counts for Sanderlings and Semipalmated Sandpipers, Delaware Bay 1986–1996. Values are peak day counts of each species on the New Jersey and Delaware shorelines, with the proportion of the bay total that occurred on the New Jersey shoreline. Peak-day surveys were identified independently for each species based on that year's maximum total.

**Results**

Sanderlings within the 1986–1996 window were consistently concentrated on the New Jersey shoreline. The mean New Jersey proportion across the 11 years was 0.825 (SD = 0.198), ranging from 0.418 in 1986 to 1.000 in 1991. Simple linear regression of New Jersey proportion on year did not detect a temporal trend within this window (slope = +0.012, SE = 0.020, t = 0.61, p = 0.556, R² = 0.04). Semipalmated Sandpipers within the same window were more evenly distributed between the two shorelines, with a mean New Jersey proportion of 0.442 (SD = 0.112, range 0.280–0.639). Linear regression again did not detect a temporal trend (slope = +0.014, SE = 0.010, t = 1.41, p = 0.191, R² = 0.18).

For direct comparison, we refit the shoreline-use regressions for Red Knots and Ruddy Turnstones over the same 1986–1996 window. Neither species showed a statistically significant trend in this early period. Red Knots had a mean New Jersey proportion of 0.462 (SD = 0.180) with a non-significant negative slope (−0.023, p = 0.184, R² = 0.19). Ruddy Turnstones had a mean New Jersey proportion of 0.484 (SD = 0.063) with a near-zero slope (+0.003, p = 0.691, R² = 0.02). The pronounced Red Knot shift toward the New Jersey shoreline documented in Figure 5 and Table 5 of the main text emerged after 1996 and is therefore not detectable in any species from the 1986–1996 clean-data window alone.

**Interpretation**

Two descriptive patterns emerge from this restricted analysis. First, Sanderlings at Delaware Bay have historically shown strong preference for the New Jersey shoreline; in seven of the 11 clean years, more than 90% of the bay-wide Sanderling population was on the New Jersey side. This asymmetry predates the shoreline redistribution described in the main text for Red Knots and reflects the historical association of Sanderlings with the sandier, wider beaches of the New Jersey shore (Villard et al. 2015). Second, Semipalmated Sandpipers used both shorelines more evenly, consistent with their broader habitat associations and their known use of interior tidal flats and salt marshes as well as beach habitats (Hicklin & Gratto-Trevor 2020).

Since the clean-data window closes in 1996 and the redistribution shift documented for Red Knots emerged after 2010, these data cannot address whether Sanderlings or Semipalmated Sandpipers underwent shoreline-use shifts comparable to the Red Knot pattern. Answering that question would require standardized reprocessing of the PEEP and MED/PEEP aggregate categories from 1997 onward, which is beyond the scope of this study. We present these results only to document that the exploratory analyses referenced in the main text (Methods 2.2) do not, on their face, contradict the main manuscript's decision to restrict the shoreline-use analysis to Red Knots and Ruddy Turnstones.

**References cited in Appendix S2**

Hicklin, P., & Gratto-Trevor, C. L. (2020). Semipalmated Sandpiper (Calidris pusilla), version 1.0. In Birds of the World (A. F. Poole & F. B. Gill, Editors). Cornell Lab of Ornithology, Ithaca, NY, USA.

Villard, P., McKinnon, L., Descamps, S., & Bêty, J. (2015). Sanderling (Calidris alba), version 1.0. In Birds of the World (S. M. Billerman, Editor). Cornell Lab of Ornithology, Ithaca, NY, USA.
