# Supplementary material for: Long‐Term Population Trends and Spatial Redistribution of Shorebirds at a Critical Migratory Stopover
Source: Ecol Evol. 2026 Sep 2;16(9):e74183. doi: 10.1002/ece3.74183 (PMC13539063; doi:10.1002/ece3.74183)
Supplement: Supplementary file 2 [file ECE3-16-e74183-s002.docx]

**Supplementary Material:**

**S Table 1:**

| **NJ Lower Bay Beaches** | | **NJ Upper Bay Beaches** | | **DE Upper Bay Beaches** | | **DE Lower Bay Beaches** | |
| --- | --- | --- | --- | --- | --- | --- | --- |
| **Seg.** | **Name** | **Seg.** | **Name** | **Seg.** | **Name** | **Seg.** | **Name** |
| 1 | Town Bank | 25 | Maurice River | 49 | Woodland Beach | 65 | South Pickering Beach |
| 2 | WW Highlands Beach/Cox Hall Creek | 26 | Elder Point | 50 | Fraland No | 66 | Kitts Hummock |
| 3 | Villas | 27 | Kenny Point | 51 | Fraland Beach | 67 | South Kitts Hummock |
| 4 | Fishing Creek/North Miami Beach | 28 | Dividing Creek | 52 | Fraland Beach | 68 | South Bowers Beach |
| 5 | Sunray Beach Preserve | 29 | Northeast Egg Island | 53 | Long Bar Point | 69 | Bennetts Pier |
| 6 | North Norbury's Landing | 30 | Egg Island Point | 54 | North Thrumcap | 70 | South Bennetts Pier (Clark Point) |
| 7 | Highs Beach | 31 | Brothers | 55 | Thrumcap | 71 | Big Stone Beach |
| 8 | North Pierce's Point/Dias Creek | 32 | False Egg Island Point | 56 | Goose Point | 72 | Conch Bar |
| 9 | South Kimbles Beach | 33 | Fishing Creek | 57 | South Leipsic | 73 | Mispillion Harbor |
| 10 | Cooks Beach | 34 | Raybins Beach | 58 | Simons River | 74 | North Slaughter Beach |
| 11 | Reeds Beach | 35 | Fortescue | 59 | Deepwater | 75 | South Slaughter Beach |
| 12 | North Reeds Beach | 36 | Beadon Point | 60 | Port Mahon River | 76 | Fowler's Beach |
| 13 | North Bidwell Creek | 37 | North Beadon Point/Sow & Pigs Creek | 61 | South Port Mahon | 77 | Prime Hook |
| 14 | Goshen Creek | 38 | Dyers Cove | 62 | Little River | 78 | Broadkill Beach |
| 15 | Dennis Creek | 39 | Gandys Beach | 63 | South Little River | 79 | Roosevelt Inlet |
| 16 | North East Creek | 40 | Nantuxent Creek | 64 | Pickering Beach | 80 | Lewes Beach |
| 17 | West Creek | 41 | Bay Point |  |  | 81 | Cape Henlopen |
| 18 | East Moores Beach | 42 | Tarpon Cove/Bach Creek |  |  |  |  |
| 19 | Moores Beach | 43 | Ben Davis Point |  |  |  |  |
| 20 | West Moores Beach | 44 | Oyster Gut |  |  |  |  |
| 21 | South Thompsons Beach/Riggins Ditch | 45 | Sea Breeze |  |  |  |  |
| 22 | Thompsons Beach | 46 | North Sea Breeze/Midmarsh Creek |  |  |  |  |
| 23 | North Thompsons Beach | 47 | Cohansey Cove |  |  |  |  |
| 24 | East Point | 48 | Cohansey Point |  |  |  |  |

Table 1. Aerial survey segment names for shorebird peak count surveys at Delaware Bay, USA, during the May spring stopover, 1986–2025. The 81 segments are divided across four geographic regions (Figure 1). This table is paired with Figure 1.
